# Water Quality Monitoring in Developing Countries; Can Microbial Fuel Cells be the Answer?

**DOI:** 10.3390/bios5030450

**Published:** 2015-07-16

**Authors:** Jon Chouler, Mirella Di Lorenzo

**Affiliations:** 1 Centre for Sustainable Chemical Technologies, University of Bath, Bath BA2 7AY, UK; E-Mail: J.Chouler@bath.ac.uk; 2 Department of Chemical Engineering, University of Bath, Bath BA2 7AY, UK

**Keywords:** microbial fuel cell, microbial sensors, toxicant, BOD

## Abstract

The provision of safe water and adequate sanitation in developing countries is a must. A range of chemical and biological methods are currently used to ensure the safety of water for consumption. These methods however suffer from high costs, complexity of use and inability to function onsite and in real time. The microbial fuel cell (MFC) technology has great potential for the rapid and simple testing of the quality of water sources. MFCs have the advantages of high simplicity and possibility for onsite and real time monitoring. Depending on the choice of manufacturing materials, this technology can also be highly cost effective. This review covers the state-of-the-art research on MFC sensors for water quality monitoring, and explores enabling factors for their use in developing countries.

## 1. Freshwater Security

The provision of safe and secure water and adequate sanitation is critical to improve livelihood security, economic growth, and to reduce health risks and vulnerability in communities. It has therefore been described as a key target within the Millennium Goals [1,2,3]. Currently, more than 700 million people lack access to safe water, and 2.5 billion do not have access to adequate sanitation. Unsafe water and poor sanitation systems lead to dehydration, malnutrition, and easily preventable diarrheal diseases, which cause over 1.6 million deaths per year. More than 99% of these water-related deaths are concentrated in developing nations, where 84% of those with no access to drinking water live in remote areas [4,5].

Providing freshwater is no simple task. The quality of water systems is affected by changes in nutrients, sedimentation, temperature, pH, and by a multitude of trace compounds, such as heavy metals, non-metallic toxicants, persistent organics and pesticides and biological factors [6,7]. More than one third of the Earth’s accessible renewable freshwater is used for agricultural, industrial and domestic purposes, which in turn leads to water contamination via a diverse range of synthetic and natural chemicals. In particular, in developing regions, such as South-East Asia and Africa, fluoride and arsenic are compounds of major concern [8]. In India alone, it is estimated that 66 million are at risk due to high fluoride content in groundwater and over 10 million due to excess arsenic [9].

## 2. Current Approaches to Water Quality Monitoring in the Developing World

### 2.1. Detection of Chemicals

As well as sourcing freshwater in developing nations, it is imperative to guarantee the safety of the water for consumption. Water quality monitoring is therefore an important part of providing safe water and improving subsequent water management [10]. Currently, a range of methods are used to test water quality, which may either be laboratory-based assessments or field test kits. Laboratory-based assessments are required when accurate detection of specific compounds must be completed. These analyses require expensive equipment at central laboratories. For example, arsenic in water systems is commonly detected by atomic absorption spectrophotometry [11], while fluoride is typically detected using a potentiometric ion-selective electrode method or ion chromatography [12]. These analyses are off-line and require sample collection which can be a problem in developing countries if the sampling location is in a remote area. This distance between sampling site and testing location adds undue time delays and costs to the water quality monitoring process [13].

Field kit tests offer a useful alternative that provides onsite water monitoring. These kits are generally used for basic analysis such as water temperature, transparency and pH. The detection of specific contaminants by onsite tests is however more difficult. The assessment via field based methods for some common contaminants is shown in Table 1. Although the detection limits are good, ranging from 2–1000 ppb for arsenic for instance, the analytical quality control of these tests may be questionable and their reproducibility are often limited too [14]. The costs of field based tests may vary widely too, from as low as ~$0.5 up to ~$11.3. Considering the large amount of samples that need testing before a water source can be safely consumed, and the relatively large amount of samples needed for frequent monitoring, these tests can also become costly and unpractical [15]. The requirement of a power source for some field-test kits, such as for colorimeters, can also be a problem in remote and rural areas.

**Table 1:** Commercially used field based test methods for common toxicants.

Toxicant	Threshold Value * (µg·L^−1^)	Method	Detection Limit (µg·L^−1^)	Approx. Cost ($ per test)	Test Time (mins)	Source/Company
Arsenic	10	Merckoquant test strip Wagtech Arsenator Hach EZ test kit ITS EconoQuick Apryon Tech Arsenic test kit	20–500 0–1000 10–500 10–1000 5–800	0.5 2.5 0.6 0.6 1.50	40 40 20 15 30	Merck Millipore Wagtech Projects [16] [16] Apryon Tech.
Cadmium	3	Wagtech Metalyser HM 1000 Merckoquant test strip	5–1000 2–500	11.3 1.4	10 15	Wagtech Projects Merck Millipore
Fluoride	1500	Wagtech Potakit(r) Merckoquant test strip HANNA Instruments colormetric	0–1500 150–800 0–20,000	6.6 2 1.4	40 15 15	Wagtech Projects Merck Millipore HANNA Instruments
Lead	10	Wagtech Metalyser HM 1000 Merckoquant test strip	5–1000 20,000–500,000	11.3 1.4	10 15	Wagtech Projects Merck Millipore
Nitrate (ion)	50	Wagtech Potakit(r) Merckoquant test strip HANNA Instruments test kit	0–20,000 0–20,000 0–50,000	6.6 1.4 0.5	40 15 10	Wagtech Projects Merck Millipore HANNA Instruments
Nitrite (ion)	3	Wagtech Potakit(r) Merckoquant test strip HANNA Instruments test kit	0–20,000 0–20,000 0–1000	6.6 1.4 0.5	40 15 10	Wagtech Projects Merck Millipore HANNA Instruments

* as recommended by [17].

### 2.2. Bioassays

Traditional chemical and physical tests for contaminants in water must often be coupled with biological methods (bioassays) to assess their biological availability and bio-toxicity and, consequently, to evaluate their potential effects on human health and the aquatic biota. These assays involve the surveying and measurement of responses from biological organisms to water sources [18]. Biological testing can also determine the effect of bioaccumulation of contaminants over long periods of time, thus giving important indications on the effects of prolonged exposures.

Traditional bioassays involve the use of bacteria as well as complex organisms, such as fish, daphnia, and algae. The responses of these organisms to chemical and physical disturbances and environmental strains is observed during a defined period of time and used as a direct indicator of the safety of the water source [19]. Bioassays are particularly useful in differentiating between biologically active and inactive isomeric molecules. They can also be used to detect very small amounts of compounds in a water body, which proves useful for understanding dose effects of a compound [20]. Moreover, bioassays can give an understanding of the combined effects of multiple contaminants in water (co-contamination). Nonetheless, these assays present critical limitations. Firstly, the response of the organism may be affected by their natural cycles (e.g., life stage, reproduction cycle), with the consequence of generating data difficult to interpret and to reproduce. Most bioassays also require long incubation times (in the order of days to weeks) and hence are not viable for onsite monitoring [21].

## 3. Biosensors and the Potential of Microbial Fuel Cell-Based Sensors

### 3.1. Biosensors for Water Quality Monitoring

The development of biosensors in recent years has opened up great perspectives to the onsite, simplified and cost-effective monitoring of water quality. In a biosensor, a biological recognition element is combined with a physical transducer to convert the biological response to a signal that depends on the analyte concentration [22]. Biosensors can be compact, relatively inexpensive and potentially disposable. They can also allow onsite monitoring, thus eliminating the costs associated with collecting, isolating, packaging and transporting the sample to be analysed, as well as providing timely readings [23].

Large proportions of biosensors are enzymatic and operate via electrochemical means. Enzymatic biosensors have the advantage of high selectivity towards the target analyte [23]. They suffer, however, from time consuming and costly enzyme purification and immobilization protocols, and short life time and poor stability, due to enzyme deactivation or leaching [24]. The use of bacteria offers instead the advantage of great simplicity associated with biocatalyst preparation, especially when large quantities are required. Microbial biosensors are also more versatile and sensitive to a large variety of analytes, thanks to the consortium of enzymes that they contain in their cells [24]. Electrochemical approaches, *i.e.*, amperometry, potentiometry, and conductometry, are usually implemented for microbial sensors [25]. Optical microbial biosensors are, however, also common [26].

Microbial biosensors have been investigated mainly as water quality monitoring devices and currently, few prototypes used as water toxicity sensors have been commercialised [25,26]. The use of microbes that survive under highly alkaline, acidic, high temperature, and saline conditions opens up attractive perspectives on water monitoring for industrial process waste monitoring [27]. The full deployment of microbial biosensors is however faced with various challenges. These include low selectivity, low detection limits, risk of contamination with other microorganisms, and mass transfer limitations caused by the necessary permeation of substrates and products through the cells [20,27].

### 3.2. Principles of MFC Technology

**Figure 1:**
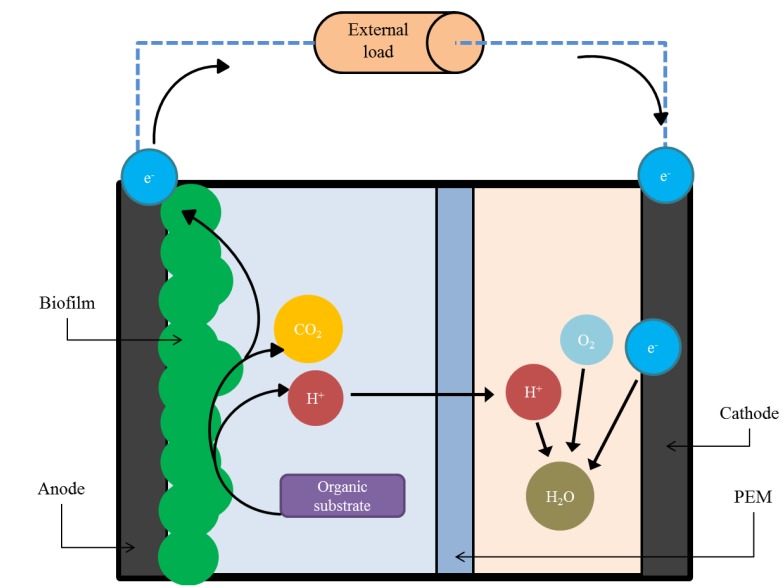
Operating principles of a two-chamber microbial fuel cell (not to scale). The electroactive biofilm at the anode break down an organic substrate to produce electrons, protons and CO_2_. The electrons pass through an external load to be reduced at the cathode.

Microbial fuel cells are devices that directly convert the chemical energy in organic matter into electricity via metabolic processes of microorganisms [28]. An MFC comprises of two electrodes, an anode and a cathode, in the presence of an electrolyte. The two electrodes are usually divided by a proton exchange membrane (PEM), and are connected by an external circuit that includes an external load (Figure 1). Electroactive bacteria (anodophiles) reside at the anode of the device in the form of a biofilm. The anodophiles oxidize the biodegradable organic molecules present in the feed solution and generate electrons, protons and carbon dioxide. In the absence of oxygen, the electrons are extracellularly transferred to the anode and flow through the external circuit towards the cathode thus producing electricity. Protons migrate through the PEM to the cathode and react with electrons and an electron accepter (usually oxygen) to form water.

Usually, carbon-based materials are implemented as electrodes. These are typically in the form of carbon cloth, carbon paper, graphite rods, plates, granules, and reticulated vitreous carbon [29]. The anode material must be porous and have a large surface area to accommodate biofilm growth. The cathode is usually doped with catalysts, such as platinum, in order to increase the rate of oxygen reduction reactions at the electrode surface. The most typically used PEM are made from Nafion^®^ or Ultrex^®^. Figure 1 shows the two-chamber configuration, which is the simplest form of MFC. Single chamber devices with the cathode, directly exposed to air as an oxygen source and a membrane bound to the cathode, are also very common. The air-cathode configuration can lead to a more compact and simpler device. The costs of operation are also reduced due to the catholyte pumping and air/oxygen purging not being required.

**Figure 2:**
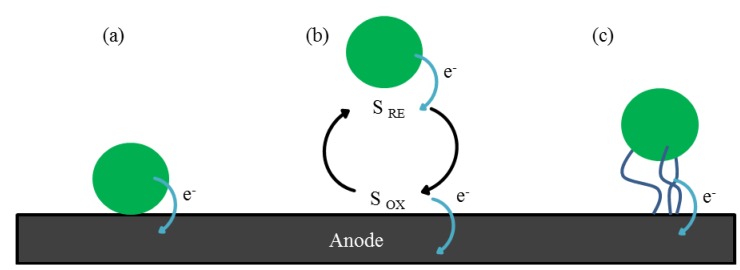
A schematic of three electron transfer mechanisms of microbes at the anode surface: (**a**) direct transfer by contact; (**b**) indirect electron transfer by redox shuttles (S _RE_ = reduced electron shuttle, S _OX_ = oxidized electron shuttle); (**c**) electron transfer by conductive nanowire matrix.

Electron transfer from the biofilm to the anode surface may occur by direct electron transfer (DET), via either direct contact or nanowires, or by mediated electron transfer (MED), which involves the use of exogenous and/or endogenous mobile electron shuttles (Figure 2) [30,31]. Bacteria, such as *Shewanella* species, can use either of these mechanisms and are therefore defined as “true anodophiles”. *Pseudomonas* species instead can only transfer electrons via a MED process involving endogenous compounds such as phenazines [32]. Examples of exogenous chemical mediators are neutral red or athraquinone-2,6-disulfonate. These are added to the anodic side to enable electron relay by bacteria that would usually be unable to transfer electrons to the electrode. The use of exogenous mediators is however not suitable for practical applications of MFCs since the cost of operation increases and possible toxicological problems of mediator release or treatment arise [20].

The anodophiles in MFCs can degrade a multitude of organic molecules in wastewater, such as acetate, propionate, butyrate [33], while simultaneously generating electricity [34,35]. The most intuitive use of the MFC technology regards therefore the development of devices that treat wastewater whilst generating electricity [36]. MFCs are in particular considered as an energy conversion technology complementary to anaerobic digesters [34,37]. Against conventional anaerobic digestion, MFC technology has the distinct advantage of treating waste with low concentrations of organics (*i.e.*, low chemical oxygen demand, COD) and at low operational temperatures (below 20 °C) [37].

Niche applications of MFCs have also been considered. The most promising regards its use as a sensor for water quality [26,38]. Given its simplicity and potential cost-effectiveness, MFC-based sensors can be the answer to effective water sensing in developing countries. So far, the use of MFCs for the measurement of the biological oxygen demand (BOD) of water has been proved [39,40,41]. There are also some preliminary encouraging applications as toxicity sensors [42,43,44].

### 3.3. MFCs as Biosensors: Operating Principles and Concepts

MFC biosensors are an avenue towards simple and sustainable monitoring for target analytes in water [45] that can be operated *in situ* and online.

**Figure 3:**
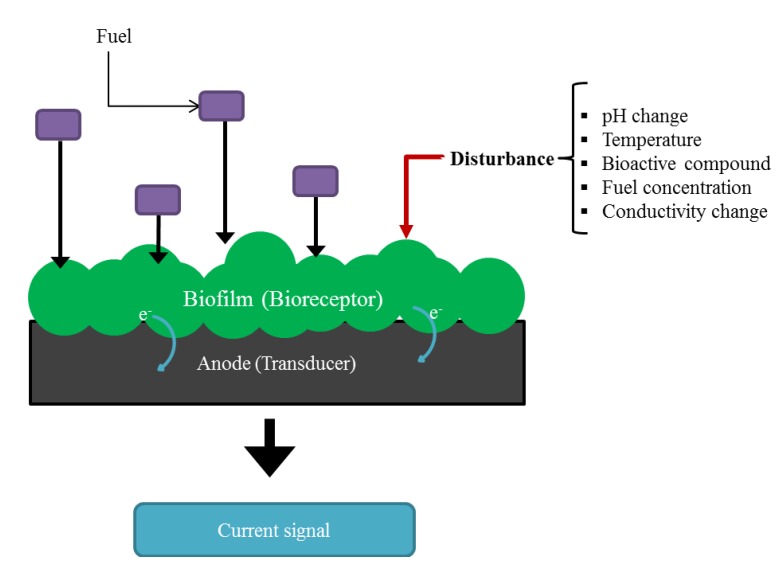
Basic principle of an MFC as a biosensor.

The current generated by an MFC directly relates to the metabolic activity of the electroactive biofilm at the anode surface [46]. Any disturbances of their metabolic pathways are translated into a change in the production of electricity. If operational parameters such as pH, temperature and conductivity of the feeding solution are kept constant, this current change can be correlated to the specific disturbance applied [26,33]. This is the basic principle behind the use of MFCs as electrochemical microbial biosensors. The anodic biofilm of the MFC acts as the recognition component (bioreceptor). Its response to the specific disturbance affects the rate of flow of electrons to the anode (the transducer) and it is transduced into a measurable current change, Figure 3. While in other types of amperometric biosensors for a substrate/analyte oxidation an external voltage has to be applied for proper biosensor function, in MFCs the driving potential is secured by a coupled oxidation of fuel on the anode surface and reduction of an oxidant on the cathode surface [47].

Providing that the anodic reactions are the limiting step, under non-saturated fuel conditions, an alteration in the concentration of biodegradable organic matter fed to the system will result in a direct change in the amount of electrons transferred to the anode and will thus cause a change in the output current [46]. Under these operating conditions, MFCs may be used as biosensors for the monitoring of the labile organic carbon in water [41]. Conversely, when the MFC is operated under saturated fuel conditions, with all other environmental factors such as temperature, pH, salinity and anode potential kept constant, a sudden change in the output current may be attributed to the presence of a bioactive compound in the feed stream [48]. The MFC technology could therefore be used to detect toxicants and biologically active components in water.

In developing the MFC technology for sensing purposes some key requirements must be met, which may differ from those associated with its use for energy harvesting. When the purpose is to generate electricity, the focus is on maximising the power output and fuel efficiency. To be used as a biosensor, the MFC must show high sensitivity towards the compound to be detected with minimal risks of false positive or negative alarms. The sensitivity is defined as the electrical signal change per unit change of analyte concentration and is usually referred to the anode surface area, according to Equation (1) [46].

(1) $$sensitivity=\;\frac{\Delta I}{\Delta c}\frac{1}{A}$$

where ΔI (μA) is the unit change in the current output; Δc (mM) is the unit change in the analyte concentration; and A is the electrode surface area (cm^2^).

High sensitivities are therefore associated with large current changes per unit change in the concentration of the target toxicant.

It is also very important that the sensor generates a constant and stable current output (baseline) [48]. In this regard, Stein *et al.* have suggested to carefully control the anodic overpotential and pH of the feed solution to the MFC, whilst maintaining substrate concentration to a saturated level [48]. In this particular study, anode potentials between −0.4 V and −0.35 V *vs.* Ag/AgCl provided the most stable output current density. The long-term stability of MFC biosensors should also be further tested.

The MFC biosensor outputs should also be reproducible and independent by operational factors, such as changes in pH, temperature and conductivity of the water samples [20]. The response time, usually defined as the time required to achieve 95% of the steady state current response, should be as short as possible. The recovery time, e.g. the time required to recover from the disturbance applied, should also be fast and the original baseline current should be fully recovered after the toxic event.

To interpret the MFC sensor outputs, the use of artificial neural networks (ANN) was suggested [43]. ANN are a form of flexible mathematical model that are used to identify complex nonlinear relationships between input and output data sets. Acetate, butyrate, glucose and corn starch were able to be correctly identified by ANN in an MFC operated under batch mode [43]. This study therefore provides a good approach for the identification of target compounds from a given MFC signal response. However, no relationships between compound concentration and signal response were established for the chemicals studied.

The advantages of MFCs over other biosensors rely on their mechanical and electrical simplicity in both design and operation. No external transducers are required to convert the biological response into a signal, as the presence of a pollutant in the feeding stream is immediately detected by a distinct current change from the system. Although the use of pure cultures has been reported [49], mixed cultures of naturally available microorganisms are usually implemented. The use of mixed cultures guarantees greater stability and it has also shown to lead to MFC-biosensors with better performance [50]. There is no need for time-consuming immobilisation procedures of the bioreceptor, as the electroactive biofilm is spontaneously formed onto the biocompatible surface of the anode during the enrichment [51]. MFC-based sensors have been shown to be able to operate onsite and continuously to provide real-time monitoring [50]. Furthermore, the electricity generated by the MFC leads to the idea of self-sustaining devices, thereby making them suitable for use in remote areas without access to energy [52].

### 3.4. MFCs as Sensors for the Labile Organic Carbon Content in Water

The biochemical oxygen demand (BOD) is a parameter traditionally used to quantify the degree of organic contamination in water systems. The BOD is commonly estimated with the BOD_5_ test that requires at least five days of incubation. This test is therefore not suitable for real-time monitoring where rapid feedback is required. In recent years, microbial biosensors have been shown to be a valid alternative to the BOD_5_ test for real-time and onsite measurements of the organic carbon content in water. Microbial fuel cells have been widely investigated as BOD sensors [39,40,41]. The first use of an MFC biosensor for BOD measurement was demonstrated in 1977 [53]. It was shown that the measured current from the device was proportional to the concentration of glucose-glutamic in a feed solution, with saturation reached at 400 mg·L^−1^ (100 µA). The first in field use of the MFC sensor was, however, reported only in 2003 [39]. The sensor showed stable performance for a period up to five years without particular maintenance, with good stability and a correlation between the sensor measurements and the BOD_5_. Table 2 gives an overview on the MFC-based BOD sensors in the literature, and a recent review on the use of MFCs for BOD monitoring can be found in [20]. As reported in Table 2, within a certain range, the electrical signal from the MFC biosensor is a direct indicator of the substrate concentration in the feed [54]. It can also be noted that the response time varies with the device design and it reaches its minimum (2.8 min) with a miniature single chamber device (anodic volume: 2 cm^3^). As shown in Table 2, MFC devices with either a platinum-doped cathode or a catalyst-free cathode have been reported, with no marked difference in performance. It can also be observed that usually the anode is inoculated with mixed bacteria for high substrate versatility and long-term stability.

### 3.5. MFCs as Toxicity Sensors

Recently, the MFC technology has been investigated also as sensor for the detection of toxicants in water systems, which are summarised in Table 3. The use of MFCs as toxicity sensors has been demonstrated for the first time in 2007 by Kim *et al.* who reported MFC response to contaminants such as Pb, Hg, Diazinon (an organophosphorus pesticide) and PCBs [45]. The lower detection limit was as low as 1 mg·L^−1^; while the upper detection limits were not fully identified given the short concentration ranges studied. Moreover, limited quantitative measurements were provided. The adaptation of the microbial community to toxic substances under continuous operation was highlighted as a concern, which suggests that an MFC biosensor could be used as a shock sensor for toxicants as opposed to a continuous operation mode sensor. The risk of microbial resistance to toxic substances has been raised by [42], who identified the use of immobilized or entrapped cells as a potential solution to this problem. Moreover, the continuous regeneration of the anode biofilm with pre-cultured electrochemically-active bacteria could provide another solution [45].

To model the effect of the toxic compound on the MFC performance, Stein *et al.* proposed the use of a modified version of the Butler Volmer Monod (BVM) equation [55]. In particular, the BVM model was modified to include four types of toxic responses related to the four inhibition kinetics of the enzyme involved in the biochemical and electrochemical reaction at the anode. These are the noncompetitive, uncompetitive, mixed and competitive inhibition kinetic, characterized by the kinetic parameter, K_i_. The resulting model was used to predict the optimum anode overpotential that leads to the highest sensitivity towards a specific toxicant. To verify its validity, the authors used this model to describe the polarization curves under non-toxic and toxic conditions for three concentrations of Ni (10, 20 and 30 mg·L^−1^) [55]. By identifying the kinetic inhibition type from the relative polarization curves, this model was suggested as a means to address the specificity of the MFC sensor to given toxicants. More work is however needed to support this proposition.

Several authors have investigated the effect of specific characteristics of the MFC design and operation on the device performance as a toxicity sensor. In particular, characteristics such as membrane type, external resistance, shear rate, single chamber devices and miniaturisation, have been studied. These are discussed below.

**Table 2:** Summary of analytical performance, construction and functional characteristics of MFCs used as BOD.

Microbe Assayed (Origin)	Anode	Cathode	Membrane Used	Configuration	Detection Range (BOD_5_, mg·L^−1^)	Saturation Signal	Response Time	Refs.
*Clostridium butyricum*	Pt	Carbon	Anion exchange membrane	Two chamber	10–300	120 µA	70 min	[53]
Enriched consortium (waste water)	Graphite felt	Graphite felt	Cation exchange membrane	Two chamber	2.58–206 (based on charge)	1.1 mA	0.5–10 h	[39]
Consortium (activated sludge)	Graphite felt	Graphite felt	Cation exchange membrane	Two chamber	23–100	6 mA	1 h	[40]
Consortium (activated sludge)	Graphite felt	Graphite felt with Pt	Cation exchange membrane	Two chamber	20–200	5.5 mA	5–36 min	[56]
Consortium (waste water)	Carbon paper	Carbon cloth with Pt	Cation exchange membrane	Single chamber (air breathing cathode)	38–324	286 mW·m^−2^	0.6 h	[57]
Consortium (anaerobic sludge)	Graphite granules	Carbon paper with Pt	Cation exchange membrane	Single chamber (air breathing cathode)	50–500	0.6 mA	40 min–2 h	[41]
Consortium (primary clarifier)	Carbon paper	Carbon paper with Pt	Cation exchange membrane	Two chamber	10–250	233 mA·m^−2^	40 min	[58]
Consortium (from an active MFC)	Carbon cloth	Carbon cloth	Cation exchange membrane	Single chamber (air breathing cathode)	3–164	35 µA	2.8–8.7 min	[46]

**Table 3:** Summary of the analytical performance, construction, and functional characteristics of MFCs used as toxicant sensors.

Microbe/s Assayed (Origin)	Anode	Cathode	Membrane	Toxicant-Detection Range	Baseline Signal	Response Time	Refs.
Consortium (Activated sludge)	Graphite felt	Graphite felt	Cation exchange membrane	Diazinon:1–10 mg·L^−1^ Pb: 1–10 mg·L^−1^ Hg: 1–10 mg·L^−1^ PCBs: 1–10 mg·L^−1^	0.04 mA	20 min–2 h	[45]
Consortium (from an active MFC)	Graphite plate	Graphite plate	Cation exchange membrane	Cu^2+^ 85 mg·L^−1^	1.37 A·m^−2^	50–100 min	[48]
Consortium (primary wastewater)	Graphite rod	Graphite rod	Cation exchange membrane	sulfamethaxozole 0.05–1000 μg·L^−1^ sulfadiazine 0.01–1000 μg.L^−1^ chloramine B 0.16–3.96 mg·L^−1^ Cu^2+^ 0.01–6.0 mg·L^−1^ Ag^+^ 0.02–1.0 mg·L^−1^ Pb^2+^ 0.41–12.48 mg·L^−1^ Hg^2+^ 0.83–8.33 mg·L^−1^	No Data	No Data	[42]
*Geobacter sulfurreducens* DSM 12127	Ti/Ni/Au tri-layer	Ti/Ni/Au tri-layer	Cation exchange membrane	Formaldehyde 0.1%–4%	4 µA·cm^−2^	<5 min	[59]
Consortium (from an active MFC)	Graphite plate	Graphite plate	Cation exchange membrane	Ni 10 mg·L^−1^	2.25 mA	30 min	[60,61]
Consortium (real domestic wastewater)	Carbon cloth	Carbon cloth coated with Pt	Cation exchange membrane	Cu^2+^ 5–7 mg·L^−1^	No Data	4 h	[62]
Consortium (waste-water)	Carbon cloth	PTFE treated carbon cloth with Pt	None	Cr^6+^ 1–8 mg·L^−1^ Fe^3+^ 1, 8, 48 mg·L^−1^ NO_3_^−^ 1, 8, 48 mg·L^−1^	0.10–0.12 V	5 min	[63]
Consortium (from an active MFC)	Carbon cloth	Carbon cloth	Cation exchange membrane	Cd^2+^ 0.1–100 µg·L^−1^	32.2 µA	12 min	[46]

The membrane in an MFC isolates the anode and cathode whilst facilitating the necessary proton transport for the redox reaction that generates the cell potential. It also helps preventing oxygen diffusion to the anode. Charged toxicant species may pass through, or be absorbed into, the membrane [60] and hence the selection of the membrane material may affect its performance as a sensor. The effect of the membrane implemented on the MFC biosensor response was investigated [60]. In particular, four ion selective membranes were tested: cation exchange, anion exchange, monovalent cation exchange and bipolar membranes. It was shown that the selection of the membrane type appeared to not significantly affect the sensitivity of the sensor.

The effect of the external resistance applied to the MFC on recovery time and sensitivity was investigated [61]. It was found that a low resistance increased sensitivity, and a high resistance resulted in a shorter recovery time. Moreover, the use of external resistance to control the response of the MFC to toxicants was concluded to be preferable over the method of controlling anode potential or current, due to faster recovery times experienced when only external resistance was controlled.

The shear rate influences the biofilm formation and structure, and the production of extracellular polymeric substances (EPS) by the bacteria; factors which will affect the diffusivity of toxicants and their interaction with the biofilm [64,65]. Therefore, these effects can have an impact on the MFC sensor performance. To investigate this effect, the feed flow rate of the feed, containing Cu^2+^ as a model toxicant, was altered and intermittent nitrogen during enrichment was sparged [62]. In particular, the authors analysed the relationships between biofilm density, porosity and EPS content of the biofilm on the sensitivity. EPS content is an important component of the biofilm since it impacts the structural integrity of the biofilm matrix [65]. It resulted that low flow rates, leading to biofilms with low density and high porosity, as well as low EPS content improved the sensitivity of the MFC towards Cu^2+^. Moreover, the use of intermittent sparging during enrichment was beneficial for the sensor sensitivity as it reduced the EPS content. A reduced EPS content of the biofilm is beneficial as it allows improved mass transport of ions towards the bacteria at the electrode surface.

The performance of a single chamber MFC devices for toxicity sensing has been compared with a two chamber device [66]. In this study, the toxicant events were simulated by altering the pH (by addition of HCl) of the inlet solution. The study demonstrated higher sensitivities for the case of the single chamber device. Moreover, by decreasing the hydraulic retention time the sensor sensitivity improved. Liu *et al.* reported the use of a simple single chamber batch MFC developed as a shock sensor for detection of Cr^6+^, Fe^3+^, and NO^3−^ in wastewater influents [63]. The MFC sensor was able to distinguish between toxic and non-toxic events based on voltage changes produced from the device. Notably Cr^6+^ ions produced a far greater response than Fe^3+^ ions. The NO^3−^ ions produced however little effect to the output voltage of the device. Finally, the open circuit potential of the anode was found to be related to the voltage change response of the device, indicating that the sensitivity of the sensor is dependent on the activity of the biofilm at the anode.

The use of a micro scale MFC toxicant biosensor has been demonstrated [59]. A silicon based device was designed, involving two 144 µL chambers divided by a proton exchange membrane, with two silicon plates sputter coated with a 150 nm Ti/Ni/Au tri-layer (active area 80 × 80 µm^2^) as current collectors. A solution containing potassium ferricyanide was used to assist the oxygen reduction reactions at the cathode. To operate the micro scale MFC as a toxicity sensor, the cell was set at a fixed current (1 µA—equivalent to a current density of 4 µA·cm^−2^) to ensure a stable baseline signal, and observing the changes in output voltage in order to detect the presence of a toxic compound. The effect of formaldehyde was tested in the MFC sensor, and concentrations between 0.1% and 4% *v*/*v* resulted in a complete drop in output voltage and hence an irreversible inactivation of the biofilm in the cell.

The first single chamber miniature device reported consisted of a small scale and simple single chamber air-cathode MFC fabricated by layer by layer 3D printing [46]. When the fuel cell was operated under saturated conditions, the presence of cadmium ions in the feeding solution was instantaneously detected by a measurable drop in the output current. This change was proportional to the concentration of cadmium within the whole range of concentration considered 1–100 µg·L^−1^. The dose–response relationship of the device was established, with a dynamic range of detection between 1 and 25 µg·L^−1^ and a sensitivity of 0.2 µA·µg^−1^·L^−1^·cm^−2^. Within the linear range, the changes to the electroactive biofilm were reversible, and recovery after the shock event was possible within 12 min. Variance of the data provided showed good repeatability, with a variability of MFC responses within 1.5%. This study highlighted the importance of micro-scaling an MFC sensor, where the use of microfabrication allowed enhanced sensitivity of the sensor and faster response times.

## 4. Challenges of Implementing MFC Biosensor Technology for Developing Countries

Microbial fuel cells hold great potential as simple-to-use, rapid and cost-effective sensing devices for water quality monitoring, in alternative to traditional analytical methods that are limited by high cost, long test times, and being offline. As a consequence, MFCs could provide great benefits to organizations operating in developing countries [67]. So far, it has been demonstrated that MFC sensors can be sensitive to target compounds with identifiable dynamic ranges and detection limits lower than 1 ppm, and are potentially stable over long-term operations. A number of key challenges must, however, be addressed for the practical deployment of this technology. These challenges are discussed below.

### 4.1. Simplicity of Use

MFC biosensors have the potential to provide a much simpler detection of bioactive toxicants in water than traditional chemical and biological methods. In the presence of a toxicant in the feeding solution, the MFC sends in fact an instantaneous warning that is easily detected as a change in the output current and does not require complex and expensive transducers. Although conceptually simple, the MFC response to a given toxicant can however be difficult to interpret. Little work has so far been performed on MFC data processing to transform the sensor readings into simple outputs easy to understand by non-experts. Artificial neural networks may provide an avenue towards simplified data outputs from the MFC [43].

The MFC assembly and the testing system must be straightforward and simple, thus allowing straightforward start-up and maintenance of the technology. The MFC design must therefore be simplified and single chamber air breathing cathode MFCs should be better explored for this [46]. The majority of the MFC biosensors mentioned in this review rely on a two chamber configuration where either sparged oxygen or ferricyanide are fed to the cathode as an electron acceptor. The benefits of using an air-breathing, single chamber device over a two chamber design include reduced operating costs associated with controlling a second feed solution, reduced capital costs of design [68], and a sustainable, passively-fed source of oxygen [69]. This in turn allows the assembly of the MFC device to involve fewer parts, and therefore simplify the system set-up and operation. Single chamber devices are also easier to miniaturise [70]. Miniaturisation of MFC devices paves the way for ready-made 3D printed devices that could also be easily assembled into stacks for multiple readings and/or for simultaneous detection of a range of toxicants. Although a couple of small scale single chamber air breathing cathode MFCs have been developed as MFC biosensors [46,63], the development of micro scale MFC biosensors as simple-to-manufacture and effective toxicant sensors still needs to be pursued. As well as reducing costs, miniaturisation also improves mass transport within the fuel cell, and hence any differences in concentration of analyte at the input and at the biofilm on the electrode are reduced, thus leading to a more reliable sensor [71]. Shorter distances within the MFC also allow a faster sensor response time [59]. Response times as short as 3 min [46] have been reported for miniature MFC sensors with clear advantages over current time-consuming biological methods. However, the process of miniaturisation of MFC biosensors is still in its infancy with further scope for miniaturisation available in order to enhance MFC biosensor performance [46].

### 4.2. Use of Inexpensive Materials

MFC devices may be cheap to manufacture, as they are commonly made out of plastics (such as Plexiglas) and carbonaceous materials used as electrodes [29]. The manufacturing costs can be further decreased by miniaturisation and by using 3D printing techniques [46]. Despite this, there must be an enhanced effort on MFC cost reduction for applications of MFC-based biosensors in developing countries. Both the membrane and the cathode catalyst heavily impact on the device cost. Usually MFCs employ an expensive proton exchange membrane, typically made from Nafion^®^ or Ultrex^®^, which are also difficult to source in developing regions. Some inexpensive alternative materials have been tested as membranes, such as latex condom [72], pre-fabricated latex gloves [73] and cast ceramics [74], with very promising results. Membraneless design has also been proposed, with a biofilm on both the anode and the cathode surfaces, providing promising output powers [75,76]. All these studies on alternative membranes are focused on MFC applications such as energy generation, but the use of such materials for sensing purposes has not been demonstrated yet.

The cathode electrode in MFCs is often doped with expensive and precious metals (e.g., Pt), although prototypes with catalyst-free cathodes have been reported [46]. Recently, the use of bio-based catalysts which are recovered from waste and applicable for use as catalysts has been suggested as means to obtain the optimal performance achieved with a Pt-cathode, whilst reducing device cost and its carbon footprint. In particular, biochars derived from wood [77], sewage sludge [78], and bananas [79] have been shown to function as effective catalysts in MFC devices for the purpose of energy generation. The full potential of these biomass-derived catalysts has not been fully exploited yet and their possible benefit in enhancing sensing performance of MFCs has not been investigated.

### 4.3. Onsite Capability

Although many studies have regarded the use of real wastewater [45,62,63], the possibility to operate the MFC technology in field has still to be fully proven. Kim *et al.* have installed an MFC biosensor into a wastewater treatment plant effluent line, which contained a consortium of substances such as aluminium, zinc, mercury and arsenic [45]. To the best of the authors’ knowledge, this is so far the only in field study reported. Even in this case, however, the shock event was mimicked by manually introducing into the feed solution a cadmium and lead mixture. The results show great promise for MFC performance on site; however, it is necessary to better investigate the sensor behaviour in real contexts.

The effect of a consortium of toxicants in real water supplies on MFC sensing performance must be identified. MFC biosensors must be able to identify toxicants within a mixed contaminant environment and still present a simple and easy to understand output signal. Stein *et al.* have suggested to act on the anode overpotential as a way to tune the sensitivity of an MFC biosensor towards specific bioactive compounds [61]. In this way, the simultaneous detection of toxicants could be performed by using an array of MFCs operated at various anode potentials [46]. The principle behind this is that the MFC sensitivity and robustness is controlled not only by the mode action of the toxic component, the affinity of the toxic component and its concentration, but also by the anode overpotential at which the MFC operates [80]. For onsite capability, MFC biosensors must be compact and readily portable. MFC water sensors will need to be operated as stacks for effective water monitoring, and the miniaturisation of devices can lead to a kit easy to transport.

Onsite sensors should also be able to wirelessly transmit their outputs to a mobile device, such as a computer or a smart phone. The wireless device would comprise of three elements: a sensing unit (*i.e.*, the MFC itself), a processing unit for processing raw data to store the results (a device capable of analysing the output current response from the MFC) and a transceiver unit for sharing data with the end user [81]. The energy generated by an MFC could be used to power some of these components, thus leading to a self-sustainable device. This is especially attractive for applications in remote areas without easy access to electricity. Renewable energy supplies, such as solar batteries, could be also considered instead, and their combination with MFCs would help ensure constant operational capability [82,83]. These renewable energy conversion techniques would be an attractive alternative to conventional batteries, which are not renewable and need periodic replacements.

There is little work on conventional MFC devices being used as power sources for sensor nodes, except for a 340 mL device used to power a piezoresistive pressure sensor node [84]. Most research in this area focusses on the use of sediment based MFCs fed with terrestrial wastewater [58,82,85,86], freshwater [87] and benthic MFCs fed with seawater [88] have been used to power a wireless sensor node within a network. The power generated by these MFCs is however susceptible to the environmental conditions in which it resides. Power generation performance of a wireless MFC device can in fact be affected by the environmental temperature [87] and the pH [85]. Moreover, the power generated by MFCs is still too low (order of W·m^−3^) to be able to power alone principle components within the sensor node, such as the transceivers and controllers [83]. In order to reliably provide the correct amount of electricity and voltage elevation to the device, an intelligent power management system must be utilized [58,87] A power management system stores energy from the MFC device and converts it into power that is high enough to operate the wireless sensor node [86], and may include such components as capacitors, charge pumps, and DC-DC converters [89].

## 5. Conclusions

The developing world is challenged with providing safe water and adequate sanitation for its population. Effective water quality monitoring methods are required in these areas that are low cost, simple to use, rapid and have onsite capability. Microbial fuel cell technology is a very promising technology with the potential to satisfy this need, especially given their recent development as sensitive and small scale devices. Research is, however, still in its infancy. In order for MFCs to be fully realized for water quality monitoring in developing countries, research must focus on: (1) producing low cost and easy to manufacture devices by using inexpensive electrode and membrane materials—ideally the cost should be less than $0.50 per test to contend with existing testing methods; (2) MFCs must be tested as sensors in realistic environments, where the system would be exposed to a consortium of toxicants, whilst still providing a simple output response; (3) Response times of devices and device portability should be further optimized by miniaturisation; (4) A self-sustaining and wireless MFC biosensor needs to be developed to ensure fully functional onsite water quality monitoring for remote regions or areas with poor infrastructure. Addressing these challenges is not an easy task and requires clear research focus and effort. The outcome will be a powerful device that can drastically improve wellbeing and livelihoods of people living in developing countries and remote areas.
